# Downregulation of Nrf2 in the Hippocampus Contributes to Postoperative Cognitive Dysfunction in Aged Rats by Sensitizing Oxidative Stress and Neuroinflammation

**DOI:** 10.1155/2023/7272456

**Published:** 2023-02-09

**Authors:** Liang Li, Fanqing Meng, Dongliang Li

**Affiliations:** ^1^Department of Anesthesiology, Qilu Hospital, Shandong University, Jinan, China; ^2^Department of Anesthesiology, Jinan Maternity and Childcare Hospital, Jinan, China

## Abstract

Postoperative cognitive dysfunction (POCD) is a recognized clinical complication defined by a new cognitive impairment arising after a surgical procedure. Elderly patients are especially vulnerable to cognitive impairment after surgical operations, but the underlying mechanisms remain elusive. Oxidative stress and neuroinflammation in the hippocampus, a brain region involved in memory formation, are considered as major contributors to the development of POCD. Activation of nuclear factor erythroid 2-related factor 2 (Nrf2), a master regulator of endogenous inducible defense system, plays a crucial role in protecting cells against oxidative stress and inflammation by enhancing transcription of antioxidant and anti-inflammatory target genes. Here, we examined whether aging downregulates Nrf2 in the hippocampus and, if so, whether downregulation of hippocampal Nrf2 contributes to POCD in aging. Young and aged rats underwent abdominal surgery or sham operation. One week later, cognitive function was assessed, and brains were collected for molecular studies. Compared with young sham rats, aged sham rats exhibited a significant reduction in expression of Nrf2 in the hippocampus. Interestingly, the expression of Nrf2 downstream target genes and levels of reactive oxygen species (ROS) and proinflammatory cytokines in the hippocampus as well as cognitive function were comparable between aged sham and young sham rats. After abdominal surgery, young rats showed significant upregulation of Nrf2 and its target genes in the hippocampus. However, aged rats did not show changes in expression of Nrf2 and its target genes but had increased levels of ROS and proinflammatory cytokines in the hippocampus, along with cognitive impairment as indicated by reduced contextual freezing time. Moreover, upregulation of hippocampal Nrf2 in aged rats with intracerebroventricular infusion of a Nrf2 activator reduced levels of ROS and proinflammatory cytokines in the hippocampus, ameliorating cognitive dysfunction after surgery. The results suggest that aging-induced downregulation of Nrf2 in the hippocampus causes the failure to activate Nrf2-regulated antioxidant defense system in response to surgical insult, which contributes to POCD by sensitizing oxidative stress and neuroinflammation. Nrf2 activation in the brain may be a novel strategy to prevent the cognitive decline in elderly patients after surgery.

## 1. Introduction

Postoperative cognitive dysfunction (POCD) is a common complication defined by a significant decline in cognitive performance after a surgical procedure [1]. POCD can last from days to months and, in a small minority of patients, even up to 1-2 years after surgery [2]. There is robust evidence that advanced age is the single strongest risk factor for the development of POCD [3, 4]. Estimated prevalence of POCD in patients over the age of 60 is 36–41% at the time of discharge from hospital after major noncardiac surgery [4], and 25% of elderly patients show significant impairments in executive functions and/or memory functions 3 months after surgical interventions [5, 6]. The long-term impact of POCD in elderly patients is marked by impaired quality of life and increased morbidity and mortality. However, the precise mechanism underlying this aging-related vulnerability to POCD remains poorly understood.

There is an increasing appreciation that oxidative stress and neuroinflammation in the brain play a critical role in the development of POCD [1]. For example, multiple rodent models of surgery display cognitive impairment, which is accompanied by increased levels of reactive oxygen species (ROS) and proinflammatory mediators in the brain, particularly in the hippocampus that is involved in memory formation and learning [7–10]. Patients who develop POCD following surgery also have increased levels of ROS and proinflammatory cytokines in the cerebrospinal fluid, which are associated with the severity of cognitive dysfunction [11–13]. Because the peripheral immune system has several modes of communication to the brain, a peripheral challenge (such as surgery) is capable of causing de novo production of ROS and proinflammatory cytokines within the brain [14, 15]. Previous studies have shown that aged subjects are far more vulnerable to peripheral challenges and that these challenges may lead to exaggerated and long-lasting inflammatory response in the hippocampus, resulting in hippocampal-dependent memory deficits [16, 17]. Thus, a better understanding of mechanisms by which aging enhances susceptibility to surgery-induced oxidative stress and neuroinflammation may be essential for developing strategies to prevent POCD.

Nuclear factor erythroid 2-related factor 2 (Nrf2) is a master regulator of the antioxidant cellular defense system and expressed in most tissues including brain. Nrf2 regulates the physiological homeostasis of cellular redox status and responses to stress and inflammation [18–20]. Under physiological conditions, Nrf2 binds to Kelch-like ECH-associated protein 1 (keap1) in the cytoplasm. Upon exposure to ROS, Nrf2 disassociates from keap1 and then translocates to the nucleus, where it transactivates genes that contain antioxidant response element (ARE) sequences to protect tissues from oxidative damage [18–20]. In addition, Nrf2 suppresses activation of the transcription factor nuclear factor-kappa B (NFkB) to inhibit the production of proinflammatory cytokines [18–20]. Deficiency of Nrf2 has been shown to promote oxidative stress and neuroinflammation in the brain in mice in response to neurotoxicant acrylamide [21] or following cerebral hypoperfusion [22]. Aging is associated with progressive Nrf2 dysfunction, and reduced Nrf2 expression and activity have been reported in many organs in aged animals [20, 23, 24]. Nrf2 has been considered as a pharmaceutical target, and there is an increasing clinical interest in using Nrf2 activators to restore decreased Nrf2 function for therapeutic purposes in peripheral and brain disorders [25]. Here, we examined whether aging downregulates Nrf2 in the hippocampus and, if so, whether downregulated hippocampal Nrf2 contributes to oxidative stress and neuroinflammation and consequent cognitive impairment after surgery.

## 2. Methods

### 2.1. Animals

Aged (24 months) and young adult (3 months) male F344/BNF1 rats were obtained from Beijing Laboratory Animal Research Center (Beijing, China). All Animals were housed on a 12 : 12-hour light-dark cycle and were given regular rat chow and tap water. Animals were allowed to acclimate to animal facility for at least 1 week prior to the start of the experiment. All procedures were approved by the Animal Care and Use Committee at Shandong University.

### 2.2. Experimental Protocols

#### 2.2.1. Protocol I: To Examine the Effects of Aging or Surgery on Nrf2 Expression in the Hippocampus

Aged and young rats were randomly assigned to 4 experimental groups (*n* = 14 rats per group): (1) young rats underwent sham surgery; (2) young rats underwent laparotomy; (3) aged rats underwent sham surgery; (4) aged rats underwent laparotomy. Laparotomy or sham operation is described below. Four days after surgery, eight animals from each group were randomly selected to receive fear acquisition training. Six and seven days after surgery, open field test and fear conditioning test were conducted, respectively. All animals were then sacrificed, and brains were quickly collected. The whole hippocampus was dissected from the brain, and the CA1 region was isolated from the hippocampus for molecular studies. Some rats from each group (*n* = 6 rats per group) were perfused with fixative for immunofluorescent study.

#### 2.2.2. Protocol II: To Examine Whether Aging-Induced Downregulation of Nrf2 in the Hippocampus Contributes to Cognitive Decline after Surgery

Aged rats were randomly assigned to 2 experimental groups (*n* = 14 rats per group): (1) aged rats underwent laparotomy with intracerebroventricular (ICV) administration of vehicle (Veh); (2) aged rats underwent laparotomy with ICV administration of bardoxolone methyl (a pharmacological activator of Nrf2). Rats were implanted with cannulas in the lateral ventricle under anesthetized condition. One week later, these animals underwent laparotomy. Immediately after surgery, vehicle or bardoxolone methyl (0.5 nmol/kg/day) was given by continuous ICV infusion for 7 days. The dose of bardoxolone methyl and the route of administration were based on a previous study showing optimal in vivo upregulation of Nrf2 expression and activity in the brain of rodents without side effects [26]. At the end of the experiment, open field test, fear conditioning test, and brain collection for molecular studies were performed identically to protocol I.

### 2.3. Surgery

Laparotomy was performed according to a previously described method [10] that mimics major abdominal surgery in humans. Briefly, rats were anesthetized with halothane and the abdominal region was shaved and thoroughly disinfected with 70% ethanol. A 3 cm vertical incision was made approximately 0.5 cm below the lower right rib to penetrate the peritoneal cavity. After manipulation of the viscera and musculature with an index finger, approximately 10 cm of the intestine was exteriorized and vigorously rubbed for 30 s. The intestines were then put back into the peritoneal cavity. The peritoneal lining and abdominal muscle were sutured by sterile chromic gut sutures, and the skin was closed with surgical staples. Sham-operated rats were treated similarly except that no incision was made.

### 2.4. Implantations of ICV Cannula and Osmotic Minipumps

Using sterile surgical conditions under anesthesia, a cannula was implanted into a lateral cerebral ventricle (stereotaxic coordinates: 1.5 mm lateral to midline, 1.0 mm caudal to bregma, and 3.5 mm ventral of dura) as described previously [26]. The cannula was fixed to the cranium using dental cement. Animals were allowed to recover for 1 week before laparotomy. Immediately after laparotomy, an osmotic minipump (Alzet Osmotic Pump, Model# 2002) for central infusion of bardoxolone methyl or vehicle was implanted under the skin on the back of the animal and connected to the cannula with the catheter tubing.

### 2.5. Fear Conditioning Test

Fear conditioning test was performed as described previously [17]. Briefly, rats were taken from their home cages and placed in a conditioning chamber (Med Associates Inc., St Albans, VT, USA) 4 days after surgery. The chamber was equipped with a speaker, a fan, and two light bulbs in the side wall, and a shock generator was connected to stainless steel grid floor. Each animal was allowed to explore the chamber for 3 min before the onset of a 15 s tone (76 dB), followed immediately by a 2 s foot shock (1.5 mA). After the termination of the shock, animals were allowed to stay in the chamber for an additional 30 s. Three days later, all animals were tested for fear of the conditioning context (a hippocampal-dependent task) and for fear of the tone (a hippocampal-independent task). The chamber was cleaned with water and a mild detergent before each test. For the context fear test, animals were placed in the same chamber in which they were conditioned, observed for 6 min without tone or foot shock, and the freezing behavior was scored. For the auditory-cued fear test, rats were placed in an altered context without grid floor and the freezing behavior was scored for 3 min. After that, the tone was delivered, and freezing behavior was scored for an additional 3 min. Freezing, defined as the complete absence of visible movement except for respiration, is used as an index of memory in the test because freezing is a dominant defensive fear response in rodents. For each animal, freezing was presented as the percentage of the observation period.

### 2.6. Open Field Test

One day prior to fear conditioning test, open field test was performed as described previously [10]. Briefly, animals were placed in a corner of the open-field box in which the floor was subdivided into 25 blocks (9 cm square) with white stripes. They were allowed to move freely for 5 min, and the activity of each animal was recorded by a video camera connected to the Any-Maze animal tracking system (Xinruan Information Technology Co. Ltd., Shanghai, China), The number of line crossings (by crossing the line with all four paws) and rearing was scored by a researcher who was unaware of the study-group assignments.

### 2.7. Real-Time PCR

Total RNA was extracted from the CA1 region of the hippocampus using RNeasy plus mini kit (QIAGEN China Co. Ltd., Shanghai, China). cDNA synthesis was carried out using a High-Capacity cDNA Reverse Transcription Kit (Thermo Fisher Scientific, Rockford, IL, USA). mRNA expression of Nrf2 and its target gene heme oxygenase-1 (HO-1), NAD(P)H quinone oxidoreductase 1 (NQO1), and superoxide dismutase-2 (SOD2) and mRNA expression of proinflammatory cytokines tumor necrosis factor- (TNF-) *α*, interleukin- (IL-) 1*β*, and IL-6 in the hippocampus were analyzed with SYBR Green real-time PCR. Sequences for each primer pair are shown in Table 1. The QuantStudio™ 3 Real-Time PCR System (Applied Biosystems, Carlsbad, CA, USA) was used to perform real-time PCR. The quantification of mRNA expression was performed with the 2(−ΔΔCt) method. mRNA data were corrected by *β*-actin and expressed as fold change relative to Young+Sham in protocol I or to Aged+Surgery+Veh in protocol II.

### 2.8. Western Blot Analysis

Protein levels of Nrf2, phosphorylated (P)-NFkB-p65, and NFkB inhibitor IkB-*α* in the CA1 region of the hippocampus were analyzed by western blot. The CA1 region was quickly dissected from the hippocampus and homogenized in an ice-cold lysis buffer with protease inhibitor. The protein concentration was determined by a BCA Protein Assay Kit (Thermo Fisher Scientific, Rockford, IL, USA). The protein samples were separated by sodium dodecyl sulfate-polyacrylamide gels and then transferred to polyvinylidene difluoride membranes. After blocking for 1 h in 5% nonfat dry milk, the membranes were incubated with primary antibodies to Nrf2 (1 : 1000, #ab92946, Abcam, Cambridge, UK), P-NFkB p65 (1 : 800, #3033), and IkB-*α* (1 : 1000, #9242) and *β*-actin (1 : 1000, #4970) (Cell Signaling Technology, Danvers, MA, USA) followed by horseradish peroxidase secondary antibodies (Santa Cruz Biotechnology, Santa Cruz, CA, USA). The blots were washed and then subjected to chemiluminescent substrate. The density of the western bands was detected and quantified with Image Lab analysis software (Bio-Rad, Hercules, CA, USA).

### 2.9. Hydrogen Peroxide Assay

The levels of hydrogen peroxide (H_2_O_2_), a major ROS, were assessed using a commercial fluorescence Amplex1 Red Hydrogen Peroxide assay kit (Invitrogen, OR, USA) as described previously [27, 28]. Briefly, the hippocampal tissue was dissected and the CA1 region was isolated from the hippocampus. After homogenization in 50 mM phosphate buffer (pH 7.4) containing 5 mM sodium azide at 4°C for 1 min, the samples were centrifuged at 4000 rpm at 4°C for 15 min and the supernatant obtained was stored at −80°C until H_2_O_2_ measurement. Samples and H_2_O_2_ standards were measured with an EnVision multilabel reader (PerkinElmer, MA, USA) at 560 nm. The H_2_O_2_ levels in the hippocampal tissue were calculated using a standard curve and were normalized to tissue protein as determined by a BCA Protein Assay Kit (Thermo Fisher Scientific, Rockford, IL, USA).

### 2.10. Immunofluorescent Study

Rat brains were collected by perfusion fixation with 4% paraformaldehyde and postfixation for another 24 h. The brains were then equilibrated with 30% sucrose, and serial coronal sections (20 *μ*m) were cut using a cryostat. Immunofluorescent staining was performed as previously described [29]. Briefly, the sections were permeabilized with 0.5% H_2_O_2_ for 20 minutes and then blocked with 10% normal horse serum for 60 min. After washing with PBS, the sections were incubated with anti-rat CD11b primary antibody (Bio-Rad, Hercules, CA, USA) and anti-rat NeuN primary antibody (a neuronal marker, Cell Signaling Technology, Danvers, MA, USA) at 4°C overnight. The sections were then washed with PBS and subjected to fluorescence-conjugated secondary antibodies (Alexa Fluor 488 and Alexa Fluor 568, Invitrogen, Carlsbad, CA, USA) for 2 h. Fluorescent images were taken by a confocal microscope (Zeiss LSM 710; Carl Zeiss, Thornwood, NY, USA). The number of activated microglia, defined by stronger immunofluorescent staining of CD11b, the presence of a clearly enlarged soma and marked changes in the appearance of the processes [29], was counted in several 0.2 mm × 0.2 mm squares and presented as a percentage of the total number of microglia.

### 2.11. Statistical Analysis

The data were analyzed using GraphPad Prism 8.0 (GraphPad Software, San Diego, CA, USA). The Kolmogorov-Smirnov test and Levene's test were applied to verify normal distributions and equal variances, respectively. Two-way ANOVA followed by Tukey's post hoc test for multiple group comparisons was used to analyze the results in protocol I (4 experimental groups). Unpaired *t*-test was used to analyze the results in protocol II (2 experimental groups). All data are presented as the mean ± SE, and statistical significance was accepted at *P* < 0.05.

## 3. Results

### 3.1. Locomotor Function and Memory Performance after Surgery

**Figure 1:**
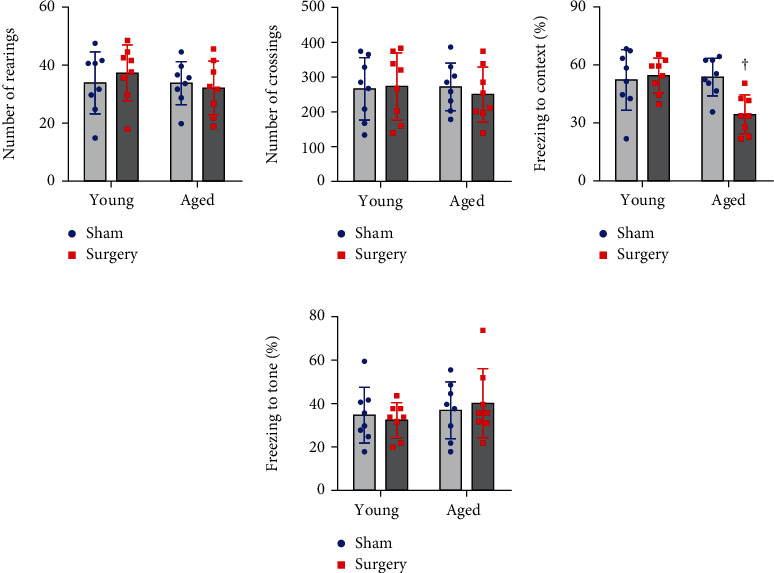
The effects of aging or surgery on spontaneous mobility and learning and memory assessed 1 week after surgery. There were no differences in (a) the number of rearings and (b) the number of crossings among four experimental groups. (c) The freezing time to the context (hippocampus-dependent memory), (d) but not to the tone (hippocampus-independent memory), as measured by fear conditioning test was reduced in the aged surgical group. Data are presented as mean ± SE (*n* = 8 rats per group) and analyzed by two-way ANOVA, followed by Tukey's multiple comparison test. ^†^*P* < 0.05 vs. the other three groups.

At the end of the study protocol, open field test was performed prior to fear conditioning test to exclude possible impairments in locomotor function that might confound the assessment of fear conditioning. It is important to note that the number of rearings (Figure 1(a)) and the number of crossings (Figure 1(b)) were similar across four experimental groups, indicating that spontaneous locomotor activity was not altered by either surgery or aging.

Two-way ANOVA revealed a significant effect of the age and a significant effect of the surgery on freezing to the context in the contextual fear conditioning test. Also, a significant interaction between the two factors for freezing to the context was revealed. Post hoc analysis showed that the freezing to the context was similar in aged sham rats and young sham rats 7 days after surgery (Figure 1(c)). However, aged but not young rats that underwent surgery displayed significantly less freezing to the context as compared to the corresponding sham group. For the auditory-cued test, there was no difference in the freezing to the tone among four experimental groups (Figure 1(d)).

### 3.2. Expression of Nrf2 and Its Downstream Target Genes in the Hippocampus

Two-way ANOVA revealed a significant effect of the age and a significant effect of the surgery on expression of Nrf2 and its downstream target genes. Also, significant interactions between the two factors for expression of Nrf2 and its downstream target genes were present.

**Figure 2:**
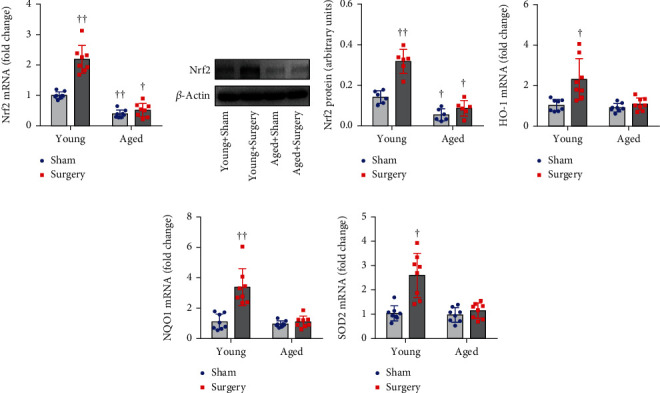
The effects of aging or surgery on (a) mRNA and (b, c) protein levels of Nrf2 and on mRNA expression of Nrf2 downstream antioxidant targets (d) HO-1, (e) NQO1, and (f) SOD2 in the hippocampus of young rats and aged rats 1 week after abdominal surgery. mRNA expression was normalized to *β*-actin and expressed as fold change relative to Young+Sham. Protein levels were normalized to *β*-actin and expressed as arbitrary units. Data are presented as mean ± SE (*n* = 6–8 rats per group) and analyzed by two-way ANOVA, followed by Tukey's multiple comparison test. ^†^*P* < 0.05 and ^††^*P* < 0.001 vs. Young+Sham.

Compared with young sham rats, aged sham rats had significantly lower mRNA (Figure 2(a)) and protein (Figures 2(b) and 2(c)) levels of Nrf2 in the hippocampus. Importantly, surgery markedly increased levels of Nrf2 mRNA and protein in the hippocampus in young rats but had no effects in aged rats.

mRNA levels of antioxidant genes HO-1 (Figure 2(d)), NQO1 (Figure 2(e)), and SOD2 (Figure 2(f)), the major downstream targets of Nrf2 in the hippocampus, were comparable between young sham rats and aged sham rats. Notably, surgery significantly increased mRNA expression of HO-1, NQO1, and SOD2 in young rats but did not change any of them in aged rats.

### 3.3. Production of ROS and Proinflammatory Cytokines in the Hippocampus

Two-way ANOVA revealed a significant effect of the age and a significant effect of the surgery on hydrogen peroxide levels and mRNA expression of TNF-*α* and IL-1*β* but not IL-6. Also, significant interactions between the two factors for hydrogen peroxide levels and mRNA expression of TNF-*α* and IL-1*β* were revealed.

**Figure 3:**
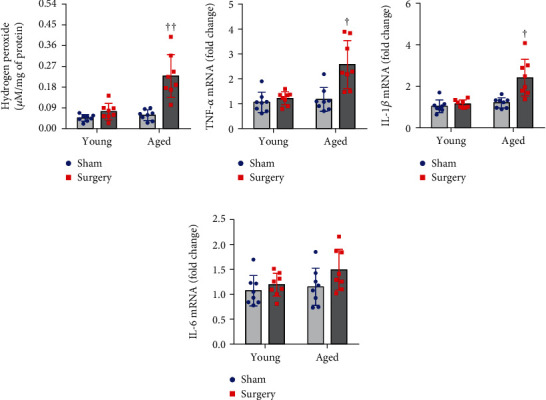
The effects of aging or surgery on (a) levels of hydrogen peroxide and on mRNA expression of proinflammatory cytokines (b) TNF-*α*, (c) IL-1*β*, and (d) IL-6 in the hippocampus of young rats and aged rats 1 week after abdominal surgery. mRNA expression was normalized to *β*-actin and expressed as fold change relative to Young+Sham. Data are presented as mean ± SE (*n* = 8 rats per group) and analyzed by two-way ANOVA, followed by Tukey's multiple comparison test. ^†^*P* < 0.05 and ^††^*P* < 0.001 vs. the other three groups.

ROS production in the hippocampus was assessed by measuring hydrogen peroxide levels. As shown in Figure 3(a), there were no significant differences in hydrogen peroxide levels in the hippocampus between the two sham groups. In young rats, hydrogen peroxide levels tended to be higher in the surgery group than in the sham group, but the difference was not statistically significant. In aged rats, hydrogen peroxide levels were markedly increased in the surgery group when compared with the sham group.

mRNA levels of proinflammatory cytokines TNF-*α* (Figure 3(b)) and IL-1*β* (Figure 3(c)) in the hippocampus were similar in aged sham and young sham rats. Surgery did not alter mRNA expression of TNF-*α* and IL-1b in the young group but significantly elevated both of them in the aged group. No significant difference in mRNA expression of proinflammatory cytokine IL-6 (Figure 3(d)) in the hippocampus was observed among four experimental groups.

### 3.4. Activation of NF-*κ*B and Microglia in the Hippocampus

Two-way ANOVA revealed a significant effect of the age and a significant effect of the surgery on levels of P-NFkB p65 and IkB-*α* and the number of activated microglia but not the number of total microglia. Also, significant interactions between the two factors for levels of P-NFkB p65 and IkB-*α* and the number of activated microglia were revealed.

**Figure 4:**
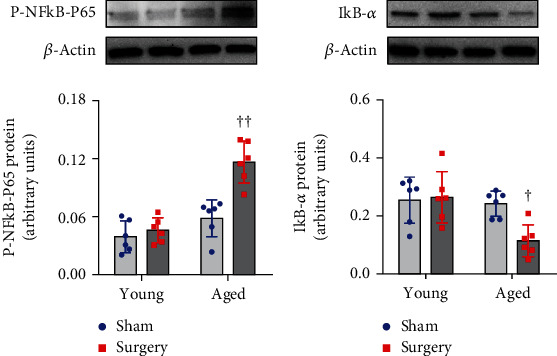
The effects of aging or surgery on protein levels of (a) phosphorylated (P)-NFkB-p65 and (b) NFkB inhibitor IkB-*α* in the hippocampus of young rats and aged rats 1 week after abdominal surgery. Protein levels were normalized to *β*-actin and expressed as arbitrary units. Data are presented as mean ± SE (*n* = 6 rats per group) and analyzed by two-way ANOVA, followed by Tukey's multiple comparison test. ^†^*P* < 0.05 and ^††^*P* < 0.001 vs. the other three groups.

There were no differences in levels of P-NFkB p65 (Figure 4(a)) and NF-*κ*B inhibitor IkB-*α* (Figure 4(b)) in the hippocampus between two sham groups. Compared with the corresponding sham group, aged but not young rats that underwent surgery had significantly higher levels of P-NFkB p65 and lower levels of IkB-*α* in the hippocampus.

**Figure 5:**
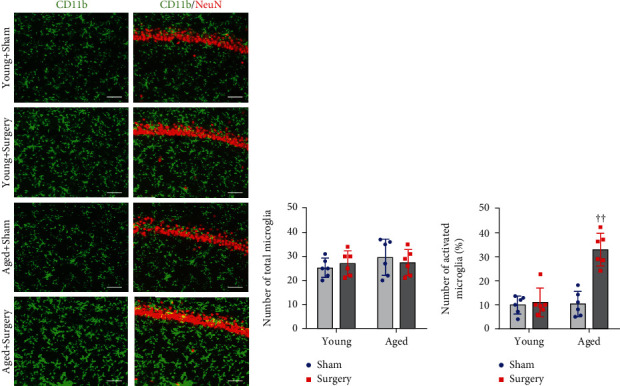
Representative photomicrographs showing (a) CD11b-immunoreactive microglia and quantitative comparison of (b) total and (c) activated microglia in the hippocampus of young rats and aged rats 1 week after abdominal surgery. NeuN: a neuronal marker. Scale bar: 50 *μ*m. Data are presented as mean ± SE (*n* = 6 rats per group) and analyzed by two-way ANOVA, followed by Tukey's multiple comparison test. ^††^*P* < 0.001 vs. the other three groups.

Immunofluorescent study showed that the number of total microglia in the hippocampus was comparable among four experimental groups (Figures 5(a) and 5(b)). The number of activated microglia in the hippocampus in aged sham rats was not different from that in young sham rats (Figures 5(a) and 5(c)). Only aged surgical rats exhibited a significant increase in the number of activated microglia compared with aged sham or young animals.

### 3.5. Effects of Central Nrf2 Activator on Cognitive Dysfunction in Aged Rats after Surgery

Given that aged rats had downregulation of Nrf2 in the hippocampus and exhibited cognitive decline following surgery, we next examine whether upregulation of Nrf2 in the brain by ICV infusion of Nrf2 activator bardoxolone methyl would ameliorate cognitive dysfunction in aged rats after surgery.

**Figure 6:**
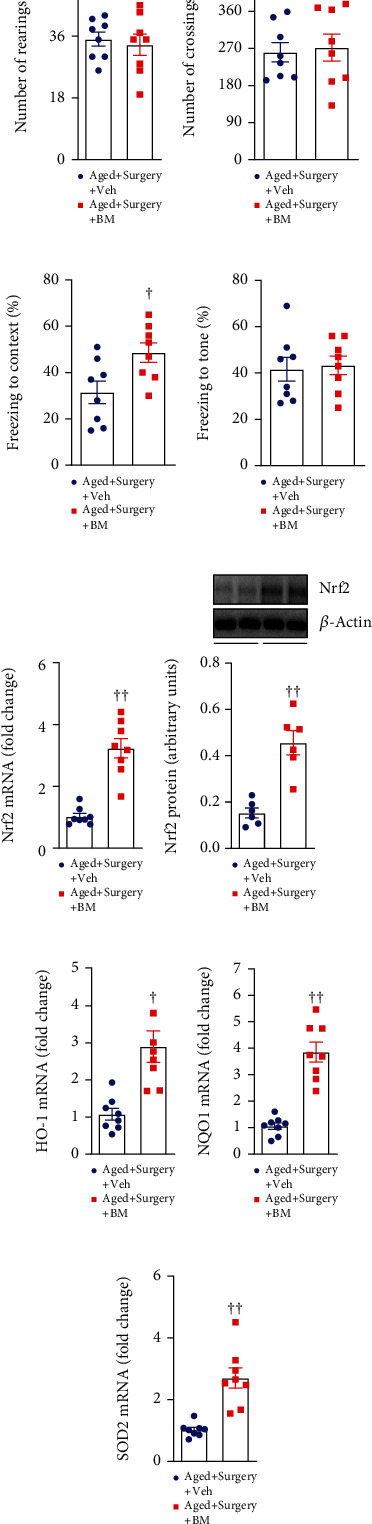
Spontaneous mobility as measured by (a) the number of rearings and (b) the number of crossings in open field test, learning and memory as measured by the freezing time to the (c) context and (d) tone in the fear conditioning test, and (e) mRNA and (f) protein levels of Nrf2 and mRNA expression of Nrf2 downstream antioxidant targets (g) HO-1, (h) NQO1, and (i) SOD2 in the hippocampus, in aged surgical rats treated with Nrf2 activator bardoxolone methyl (BM) or vehicle (Veh). mRNA expression was normalized to *β*-actin and expressed as fold change relative to Aged+Surgery+Veh. Protein levels were normalized to *β*-actin and expressed as arbitrary units. Data are presented as mean ± SE (*n* = 6–8 rats per group) and analyzed by unpaired *t*-test. ^†^*P* < 0.05 and ^††^*P* < 0.001 vs. Aged+Surgery+Veh.

As shown in Figure 6, there was no difference in spontaneous motor activity as measured by the number of rearings (Figure 6(a)) and the number of crossings (Figure 6(b)) between the two aged groups. Of note, compared with vehicle treatment, bardoxolone methyl treatment significantly increased the freezing to the context (Figure 6(c)) but did not change the freezing to the tone (Figure 6(d)) as assessed by fear conditioning test in aged surgical rats.

Bardoxolone methyl treatment, compared with vehicle treatment, markedly increased levels of Nrf2 mRNA and protein (Figures 6(e) and 6(f)). Moreover, mRNA expression of Nrf2 targets HO-1, NQO1, and SOD2 (Figures 6(g)–6(i)) in the hippocampus in aged surgical rats was also significantly elevated by bardoxolone methyl treatment. These data verified effective upregulation of the Nrf2-ARE transcriptional pathway in the hippocampus by ICV bardoxolone methyl treatment.

### 3.6. Effects of Central Nrf2 Activator on ROS Production and Neuroinflammation in Aged Rats after Surgery

**Figure 7:**
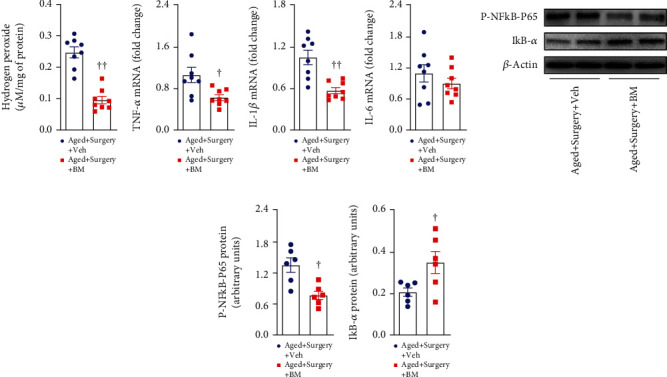
The (a) levels of hydrogen peroxide, mRNA expression of proinflammatory cytokines (b) TNF-*α*, (c) IL-1*β*, and (d) IL-6, and protein levels of (e, f) (P)-NFkB-p65 and (e, g) NFkB inhibitor IkB-*α* in the hippocampus of aged surgical rats treated with Nrf2 activator bardoxolone methyl (BM) or vehicle (Veh). mRNA expression was normalized to *β*-actin and expressed as fold change relative to Aged+Surgery+Veh. Protein levels were normalized to *β*-actin and expressed as arbitrary units. Data are presented as mean ± SE (*n* = 6–8 rats per group) and analyzed by unpaired *t*-test. ^†^*P* < 0.05 and ^††^*P* < 0.001 vs. Aged+Surgery+Veh.

**Figure 8:**
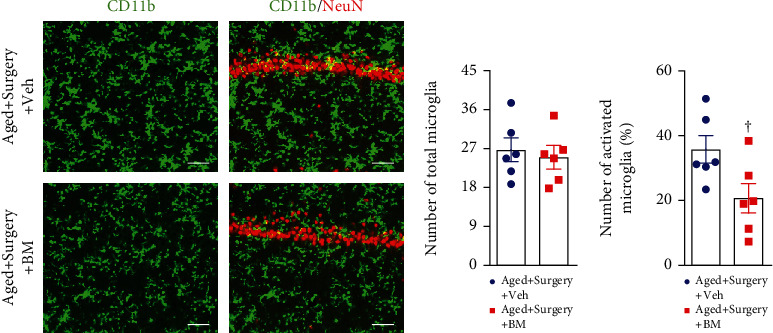
Representative photomicrographs showing (a) CD11b-immunoreactive microglia and quantitative comparison of (b) total and (c) activated microglia in the hippocampus of aged surgical rats treated with Nrf2 activator bardoxolone methyl (BM) or vehicle (Veh). NeuN: a neuronal marker. Scale bar: 50 *μ*m. Data are presented as mean ± SE (*n* = 6 rats per group) and analyzed by unpaired *t*-test. ^†^*P* < 0.05 vs. Aged+Surgery+Veh.

Compared with vehicle treatment, bardoxolone methyl treatment significantly reduced levels of hydrogen peroxide (Figure 7(a)) and mRNA expression of proinflammatory cytokines TNF-*α* (Figure 7(b)) and IL-1*β* (Figure 7(c)), which were accompanied by decreased levels of P-NFkB p65 (Figures 7(e) and 7(f)) and increased levels of IkB-*α* (Figures 7(e) and 7(g)) in the hippocampus. There was no difference in mRNA expression of IL-6 between two groups (Figure 7(d)). Moreover, the number of total microglia (Figures 8(a) and 8(b)) was unchanged, but the number of activated microglia (Figures 8(a) and 8(c)) was significantly attenuated in aged surgical rats after bardoxolone methyl treatment.

## 4. Discussion

It is now recognized that aging brain is more vulnerable to the insult of a surgical procedure resulting in cognitive impairment, but the underlying mechanisms are not well understood. Here, we examined whether aging-related deficiency in central Nrf2 sensitizes POCD in aged animals. The novel findings of the present study are as follows: (1) aged rats have downregulated Nrf2 mRNA and protein in the hippocampus when compared with young rats; (2) the levels of Nrf2 and its downstream antioxidant and anti-inflammatory defense genes in the hippocampus are significantly increased in response to abdominal surgery in young rats but not in aged rats; (3) only aged rats exhibit elevated ROS production and neuroinflammation in the hippocampus and cognitive impairment after abdominal surgery; (4) upregulation of Nrf2 in the hippocampus of aged rats with Nrf2 activator reduces ROS production and neuroinflammation, ameliorating cognitive dysfunction after surgery. Taken together, these results suggest that downregulated Nrf2 in the brain of aged rats leads to the failure of transcription for Nrf2 mediated-antioxidant and anti-inflammatory defense genes in response to surgical insult, contributing to oxidative stress and neuroinflammation in the hippocampus and consequent cognitive impairment.

We and others have consistently showed that oxidative stress and neuroinflammation in the hippocampus play an important role in the development of POCD [10, 17, 30–33]. Surgical stimulation can raise the levels of NADPH oxidase in the brain to generate ROS, leading to direct damage of neural tissues [1]. Oxidative stress can also trigger neuroinflammation by activating the NFkB signaling pathway to produce a variety of proinflammatory cytokines, which in turn promote oxidative stress. These two components create a vicious cycle of oxidative stress and inflammation enhancing each other and so resulting in cognitive impairment [1, 34]. Microglia, the key innate immune cells in the brain that can be activated by surgical insult, are the major sources of ROS and proinflammatory cytokines. The mammalian cells including microglia are equipped with endogenous inducible defense systems to neutralize excessive ROS via enzymatic and nonenzymatic compounds, most of which are regulated by Nrf2 [18–20, 25, 35]. When ROS are elevated, Nrf2 is released from keap1 in the cytoplasm and translocates to the nucleus, where Nrf2 upregulates a broad array of its target genes encoding proteins involved in antioxidation, anti-inflammation, and detoxification, such as SOD1, SOD2, catalase, NQO1, HO-1, *γ*-glutamyl cysteine ligase-catalytic, and *γ*-glutamyl cysteine ligase-modulatory [18, 19, 25]. Nrf2 is abundantly expressed in the brain including the hippocampus and has been shown to play an important role in the modulation of microglial dynamics between the proinflammatory and anti-inflammatory phenotypes [36]. Deletion of the Nrf2 gene has been demonstrated to cause the failure to upregulate antioxidant target genes of Nrf2 in the brain in response to acrylamide, resulting in increased microglia activation, oxidative stress, neuroinflammation, and enhanced neurotoxicity in mice [21]. In contrast, pharmacologic activation of Nrf2 reduces activation of microglia and neuroinflammation, alleviates neurotoxicity, and improves synaptic and mitochondrial function in animals following lipopolysaccharide challenge [37–40]. Previous studies have demonstrated that Nrf2 expression and activity are decreased in the spinal cord and carotid artery in aged animals [23, 24, 41]; our results showed that mRNA and protein levels of Nrf2 in the hippocampus were significantly lower in aged sham rats than young sham rats, suggesting that Nrf2 declines with age in the hippocampus, a key brain region involved in memory formation and spatial learning. After surgery, mRNA and protein levels of Nrf2, along with expression of Nrf2-regulated antioxidant genes in the hippocampus, were significantly increased in young rats. However, aged rats did not show significant changes in levels of Nrf2 and its target genes in response to surgery. Consequently, aged rats but not young rats had significant increases in levels of ROS, expression of inflammatory cytokines TNF-*α* and IL-1*β*, activation of NF-*κ*B and microglia in the hippocampus, and cognitive impairment following surgery. These results suggest that Nrf2-regulated antioxidant defense relies on an intact Nrf2 system in the brain. Aging-related downregulation of Nrf2 causes the failure to trigger an antioxidant defense system, leading to increased vulnerability to surgery-induced oxidative stress and neuroinflammation in the hippocampus and subsequent cognitive impairment. Interestingly, there was no significant difference in mRNA expression of proinflammatory cytokine IL-6 in the hippocampus between groups. Experimental studies in rodents have shown that traumatic brain injury induces an increase in IL-6 mRNA expression in the brain tissues after 1 h, and peak in protein levels is observed between 2 and 8 h after injury [42]. In cerebrospinal fluid, increases in IL-6 protein levels can be detected within 1 h, with peak levels between 2 and 5 h after brain injury [42]. Clinical study in patients showed that IL-6 protein levels in cerebrospinal fluid were significantly increased on days 2 and 3 after stroke and then returned to normal levels 1 week later [43]. In our study, we did not observe a significant increase in mRNA expression of IL-6 measured 7 days after surgery, suggesting that IL-6 might be an early marker of inflammatory response in the hippocampus in response to surgical exposure. It is worth noting that the basal levels of antioxidant genes, basal levels of ROS, expression of inflammatory mediators, and activation of microglia in the hippocampus are similar between two sham groups. These results indicate that other factors, such as antioxidant enzyme methionine sulfoxide reductase A or specialized proresolving mediators that also participate in cell and tissue protection [44–46], might play a dominant role in maintaining the basal level of antioxidant genes, basal levels of ROS and inflammatory mediators, and microglia activation under normal conditions. Indeed, our findings are in line with a recent study showing that knockdown of Nrf2 in mice did not alter the levels of its downstream target genes, nor expression of proinflammatory cytokines in the brain under normal conditions. However, knockdown of Nrf2 abolished the upregulation of Nrf2-mediated antioxidant genes in the brain in response to acrylamide, leading to enhanced expression of proinflammatory cytokines and neurotoxicity [21].

To further confirm the contribution of downregulated Nrf2 to surgery-induced oxidative stress and neuroinflammation and cognitive impairment in aged rats, we treated aged rats with ICV infusion of a Nrf2 activator bardoxolone methyl after surgery. We found that ICV bardoxolone methyl treatment markedly elevated mRNA and protein levels of Nrf2 in the hippocampus, which were accompanied by significant increases in Nrf2-mediated antioxidant genes and decreases in levels of ROS, expression of inflammatory cytokines, and activation of NF-*κ*B and microglia in the hippocampus of aged rats following surgery. Moreover, bardoxolone methyl treatment improved surgery-induced cognitive impairment in aged rats as evidenced by increased freezing to the context. These data provide the direct evidence that aging-induced downregulation of Nrf2 in the hippocampus accounts for POCD due to the failure to activate the Nrf2-regulated antioxidant defense system to prevent oxidative stress and neuroinflammatory response after surgery.

One limitation of the present study should be acknowledged. Expression of Nrf2 and P-NFkB p65 was assessed using total proteins instead of nuclear extracts from the hippocampus. It is known that both Nrf2 and NFkB p65 translocate to the nucleus when they are activated. Further studies are needed to verify the effect of aging or surgery on the expression of these proteins in hippocampal nuclear extracts.

In conclusion, the present study demonstrates that aging-induced downregulation of Nrf2 in the hippocampus causes the failure to activate the Nrf2-regulated antioxidant defense system in response to surgical insult, which contributes to POCD in aged rats by sensitizing surgery-induced oxidative stress and neuroinflammation. Our findings also suggest that Nrf2 activation in the brain may be a novel strategy for preventing the cognitive decline in elderly patients after surgery.

## Figures and Tables

**Table 1:** Sequences for primers.

Gene	Primers	Sequences
Nrf2	Forward primer Reverse primer	5′-GCCAGCTGAACTCCTTAGAC-3′ 5′-GATTCGTGCACAGCAGCA-3′
HO-1	Forward primer Reverse primer	5′-CGACAGCATGTCCCAGGATT-3′ 5′-TCGCTCTATCTCCTCTTCCAGG-3′
NQO1	Forward primer Reverse primer	5′-CATTCTGAAAGGCTGGTTTGA-3′ 5′-CTAGCTTTGATCTGGTTGTCG-3′
SOD2	Forward primer Reverse primer	5′-AGGAGAGTTGCTGGAGGCTA-3′ 5′-AGCGGAATAAGGCCTGTTGTT-3′
TNF-*α*	Forward primer Reverse primer	5′-AAATGGGCTCCCTCTCATCAGTTC-3′ 5′-TCTGCTTGGTGGTTTGCTACGAC-3′
IL-1*β*	Forward primer Reverse primer	5′-CACCTCTCAAGCAGAGCACAG-3′ 5′-GGGTTCCATGGTGAAGTCAAC-3′
IL-6	Forward primer Reverse primer	5′-TCCTACCCCAACTTCCAATGCTC-3′ 5′-TTGGATGGTCTTGGTCCTTAGCC-3′
*β*-Actin	Forward primer Reverse primer	5′-CAGGGTGTGATGGTGGGTATGG-3′ 5′-AGTTGGTGACAATGCCGTGTTC-3′

## Data Availability

All supporting data for this manuscript are included in the figures and available from the corresponding author on reasonable request.
